# Determinants of severity levels of anemia among pregnant women in Sub-Saharan Africa: multilevel analysis

**DOI:** 10.3389/fgwh.2024.1367426

**Published:** 2024-04-09

**Authors:** Lire Lemma Tirore, Abriham Shiferaw Areba, Habtamu Tamrat, Aklilu Habte, Desta Erkalo Abame

**Affiliations:** ^1^ Department of Public Health, Wachemo University, Hossana, Ethiopia; ^2^ Department of Orthopedic Surgery, Wachemo University, Hossana, Ethiopia

**Keywords:** severity levels, anemia, pregnant women, multilevel analysis, Sub-Saharan Africa

## Abstract

**Background:**

Anemia is a severe public health problem affecting 54% of pregnant women in SSA Yet, only a limited number of studies have provided a partial assessment of the pooled prevalence and related determinants of the severity levels of anemia in pregnant women in SSA. Therefore, this study provides the most recent estimates of anemia severity levels and related determinants.

**Methods:**

The most recent Demographic Health Survey (DHS) dataset of 21 Sub-Saharan African countries which were collected between 2015 and 2022 were used. A total of 14,098 pregnant women were included. Multilevel ordinal logistic regression was used.

**Results:**

The pooled prevalence of anemia was 51.26%. Pregnant women who were in the old age groups, and who have attended secondary and higher education were less likely to be at higher levels of anemia. Those women who have given birth to >1 children in the last 5 years, pregnant women in second and third trimester and living in poorest households had greater odds of being at higher levels of anemia.

**Conclusion:**

In Sub-Saharan Africa, anemia is a severe public health concern for pregnant mothers. When developing and implementing strategies for the prevention and control of anemia, it is imperative to take into account the individual and community circumstances. Programs for the prevention and control of anemia should incorporate the economic and educational empowerment of women.

## 1 Introduction

Anemia is a condition in which the amount and size of red blood cells, or the hemoglobin (Hb) concentration, falls below a set threshold, hence decreasing the blood's capacity to deliver oxygen throughout the body (1). A pregnant woman is considered anemic when her Hb level is less than 11 g/dl (1). Based on Hb level, anemia is categorized as mild, moderate, or severe (2).

About half (50%) of anemia in women is due to iron deficiency. Folate and Vitamin B12—deficiency, parasitic infections, malaria, chronic diseases, loss of appetite, and low dietary diversity are among other causes of anemia in pregnancy (3, 4).

Because of increased blood volume, increased body need for iron to produce additional blood for the growing fetus, poor absorption and inadequate dietary intake make pregnant women more prone to anemia (5, 6).

Anemia increases the risk of preterm birth, maternal death, low birth weight, miscarriages, and stillbirths during pregnancy. It is an indication of poor health and inadequate nutrition (7, 8). It has contributed to over 25% of maternal deaths, newborn mortality, fetal impairment, and infant death (9, 10). Anemia caused 50 million years of healthy life lost due to disability in 2019 (11).

Anemia impairs the economic productivity and development of communities and countries (7). Each individual with anemia bears an economic burden ranging from $US29, 511 to $US7, 092 (12).

Since 2000, the prevalence of anemia in pregnant women has decreased only slightly. In 2022, around 50% of pregnant women worldwide suffered from anemia. The highest prevalence of anemia is observed in low- and lower-middle income countries. Sub-Saharan Africa faces a significant public health challenge with over half (57.0%) of the pregnant women affected by anemia (13, 14). Age, education, reproductive and obstetric factors, and maternal health service usage and residency have been found to be associated with anemia (15).

As a matter of public health concern, governmental and non-governmental organizations are keeping a close eye on anemia since it affects billions of people worldwide and puts them at risk for a variety of social, psychological, and economic difficulties (16). The World Health Assembly adopted a goal in 2012 to reduce the prevalence of anemia in pregnant women by 50% by the year 2025 (17). Furthermore, the Sustainable Development Goal states that by 2030, the worldwide maternal mortality ratio should be fewer than 70 per 100,000 live births. To achieve these objectives at the regional level, reliable and effective data and monitoring systems would be necessary. It is essential to gather enough knowledge about the individual and community-level factors leading to anemia in order to design timely interventions in the prevention and treatment of anemia. Yet, only a limited number of studies have provided a partial assessment of the pooled prevalence and related factors of the severity levels of anemia in pregnant women in SSA. Therefore, using the most recent data available from the DHS of the 21 SSA nations, this study provides the most recent estimates of anemia severity levels and related determinants, which will be beneficial to public health authorities.

The health, well-being, and capacity for economic growth and community development of future generations will all benefit from addressing the variables that contribute to anemia in SSA pregnant women. This will also help to ensure that mothers and newborns have healthier pregnancy outcomes. In order to create prompt interventions for the prevention and treatment of anemia, it is imperative that sufficient evidence regarding the individual and community-level factors contributing to anemia be generated (7).

## 2 Methods

### 2.1 Data source and sampling procedure

The most recent Demographic Health Survey (DHS) dataset, which were gathered in 21 Sub-Saharan African nations between 2015 and 2022, were used in this investigation. Enhancing the collection, processing, and exchange of population, health, and nutrition data while expediting their application to planning, policy-making, and program administration is the main objective of the nationally representative DHS survey. The Inner City Fund (ICF) International gave us permission to use the datasets. The DHS website (http://dhsprogram.com) was then used to download each individual data set. The DHS report for each nation contains information about the DHS.

The datasets from 21 countries have been appended together in order to estimate the pooled prevalence and identify factors that are related to the severity levels of anemia among pregnant women in Sub-Saharan Africa. A two-stage stratified sampling approach was used select women during DHS data collection. Firstly, Enumeration Areas (EAs) were chosen with a probability proportional to EA size. Next, using an equal probability systematic selection, a fixed number of 20–28 households per EA were chosen. Next, utilizing the HemoCue fast testing technology, hemoglobin levels were measured in willingly consenting women living in the designated homes. Next, depending on the hemoglobin level, the anemic status was established. Since the fetus's iron requirements and the mother's increased blood volume during pregnancy lower blood hemoglobin levels, pregnancy-related Hb levels were modified. Altitude and smoking were also taken into consideration. A total of 14,098 pregnant women from 21 SSA countries were included in this study (18) (Table 1).

**Table 1:** The number of pregnant women from 21 SSA countries included in the study, 2024.

Country	Number of pregnant women
Burkina Faso	775
Benin	878
Gambia	499
Mali	562
Nigeria	1,525
Liberia	335
Côte d'Ivoire	626
Guianea	455
Cameroon	600
Gabon	470
Ethiopia	1,053
Burundi	664
Madagaskar	662
Malawi	632
Ruwanda	413
Sera Leone	448
Tanzania	1,124
Uganda	634
Zambia	1,073
Zimbabwe	564
Republic of South Africa	106

### 2.2 Dependent variable

Depending on the Hb level, anemia is classified as none, mild, moderate, or severe and is an ordinal dependent variable.

### 2.3 Independent variables

Owing to the DHS dataset’s hierarchical structure, the independent variables were divided into categories at the individual and community levels.

Individual-level variables include age, marital status, level of education, exposure to the media, use of tobacco products, number of births within the previous 5 years, the number of household members, wealth index, access to a toilet, distance to health facility, and type of cooking fuel.

Community-level variables include place of residence and region of residence.

### 2.4 Statistical analysis

STATA version 14 was used for data cleaning and analysis. Means, medians, and proportions were used to summarize the data. To present the data, tables and graphs were employed. Because of the hierarchical nature of the DHS data, multilevel (two-level) ordinal logistic regression was used to identify factors related to anemia severity levels.

Four different models were fitted.

The first model, known as the null model, had no independent variables and represented the variation in anemia among pregnant women that was explained by cluster differences in the absence of the explanatory variables. The second model involved individual-level variables, the third involved community-level variables, and the fourth involved a model that combined significant variables from the second and third models.

A sample weight was used in consideration of the survey's complex sampling design. The adjusted odds ratio was estimated with a 95% confidence interval. Variables in each model with a *p*-value <0.05 were taken into account for the full model. The likelihood ratio test was used to test the proportional odds assumption, and the result showed that the assumption was satisfied (*p*-value = 0.87). Akaike information criteria (AIC) was used to compare the models, and the model that had the lowest AIC was deemed to be the best fit.

## 3 Results

### 3.1 Characteristics of the participants

More than quarter (26.34%) of the pregnant women were in the age group of 20–24 years. The mean age of the pregnant women was 26.76 years (SD = 6.81years). More than one-third of the pregnant women had not attended formal education (35.35%) and 35.15 had no mass media exposure. Nearly one-fourth (24.76%) were living in poorest households and 35.32% had no birth in the last five years. More than half (52.15%) and 31.6% of the pregnant women had to access to improved toilet facility and improved water source. Majority of the pregnant women (87.27%) were living in households using clean cooking fuel and 69.02% were rural residents (Table 2).

**Table 2:** Frequency and percentage distribution of characteristics of pregnant women in SSA, 2024.

Variables	Category	Frequency	Percent
Age in 5-year groups	15–19	2,132	15.12
	20–24	3,714	26.34
	25–29	3,435	24.37
	30–34	2,603	18.46
	35–39	1,598	11.33
	40–44	526	3.73
	45–49	90	0.64
Women educational status	No education	4,984	35.35
	Primary	4,682	33.21
	Secondary	3,893	27.61
	Higher	539	3.82
Births in last 5 years	No	4,979	35.32
	One	6,375	45.22
	More than one	2,744	19.46
Marital status	Living with partner	12,463	88.40
	Not living with partner	1,635	11.60
Mass media exposure	Not at all	4,955	35.15
	Less than once/week	2,563	18.18
	At least once/week	5,429	38.51
	Everyday	1,151	8.16
Smoking cigarette or tobacco	No	13,970	99.09
	Yes	128	0.91
Type of toilet	Unimproved	7,352	52.15
	Improved	6,746	47.85
Water source	Unimproved	4,455	31.60
	Improved	9,643	68.40
Type of cooking fuel	Clean	12,304	87.27
	Solid	1,794	12.73
Number of household members	<=2	1,634	11.59
	3 or 4	4,055	28.76
	>=5	8,409	59.65
Wealth index	Poorest	3,490	24.76
	Poorer	3,003	21.30
	Middle	2,717	19.27
	Richer	2,641	18.73
	Richest	2,247	15.94
Distance to health facility	Big problem	5,647	40.06
	Not a big problem	8,451	59.94
Place of residence	Urban	4,368	30.98
	Rural	9,730	69.02

### 3.2 Prevalence of anemia in pregnant women in SSA

**Figure 1:**
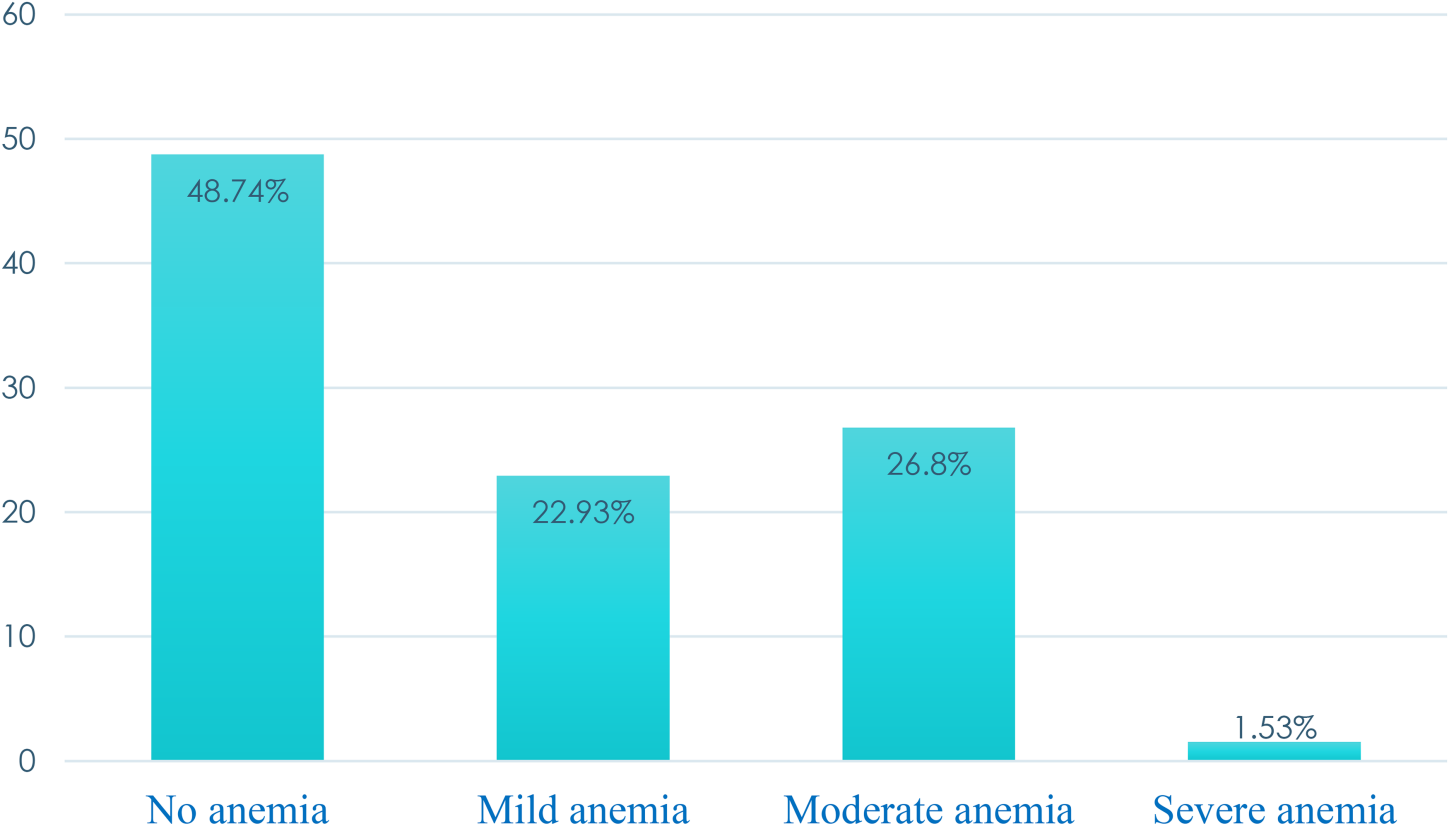
The pooled prevalence of severity levels of anemia among pregnant women in SSA, 2024.

The pooled prevalence of anemia was 51.26% with a median hemoglobin level of 10.9 g/dl (IQR: 9.8, 12 g/dl). More than one-fourth (26.8%) and one in ten (1.53%) were moderately and severely anemic, respectively (Figure 1).

**Figure 2:**
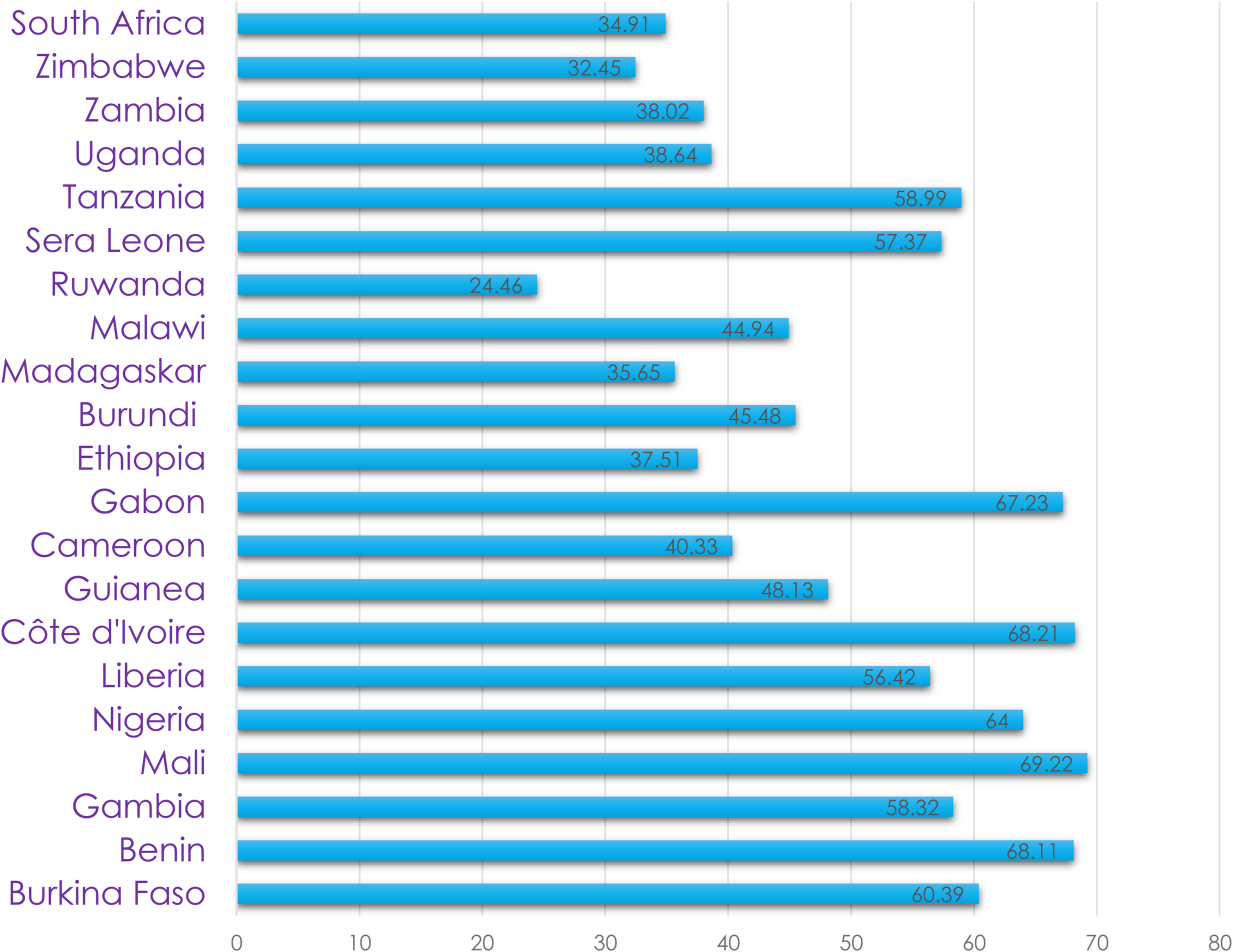
Percentage distribution of anemia among pregnant women of SSA countries, 2024.

The prevalence of anemia was the highest in Mali (69.21%) followed by Cote D'Ivoire (68.21%) (Figure 2).

### 3.3 Predictors of severity levels of anemia among pregnant women in SSA

Age, education, marital status, number of births in the last 5 years, pregnancy trimester, and wealth index, and region of residence were significantly associated with anemia.

Pregnant women who were in the age groups of 20–24, 25–29, 30–34, 35–39, 40–44 and 45–49 were 25% (AOR = 0.75), 43% (AOR = 0.57), 40% (AOR = 0.60), 47% (AOR = 0.57), 46% (AOR = 0.54), and 61% (AOR = 0.39) less likely to be at higher levels of anemia as compared to those in the age group of 15–19 years, respectively.

Pregnant women who have attended secondary and higher education had 24% (AOR = 0.76) and 52% (AOR = 0.48) lesser odds of being at higher levels of anemia, respectively, as compared to those who have not attended education. Those women who have given birth to more than one births in the last five years before survey had 16% greater odds of being at higher levels of anemia compared to women who gave no birth. The odds of being at higher levels of anemia were 4.06(AOR = 4.06) and (AOR = 4.55) times greater for pregnant women in second and third trimester, respectively as compared to those in first trimester.

Pregnant women not living with their partner had 34% (AOR = 1.34) greater odds of being at higher levels of anemia as compared with those living with their partner. The odds of higher levels of anemia were 1.82(AOR = 1.82), 1.75(AOR = 1.75), 1.40(AOR = 1.40), and 1.44(AOR = 1.44) times greater for pregnant women living in poorest, poor, middle and rich households, respectively, as compared to those living in richest households (Table 3).

**Table 3:** Determinants of severity levels of anemia among pregnant women in SSA, 2024.

Variables	Model 2 AOR (95% CI)	Mode3 AOR (95% CI)	Model4 AOR (95% CI) (final model)
15–19	1		1
20–24	0.51 (0.42, 0.62)*		0.75 (0.60, 0.92)*
25–29	0.62 (0.36, 0.82)*		0.57 (0.46, 0.72)*
30–34	0.65 (0.37, 0.85)*		0.60 (0.47, 0.75)*
35–39	0.49 (0.31, 0.78)*		0.53 (0.41, 0.68)*
40–44	0.50 (0.28, 0.88)*		0.54 (0.38, 0.78)*
45–49	0.40 (0.27, 0.98)*		0.39 (0.17, 0.88)*
No education	1		1
Primary	0.88 (0.64, 1.09)*		0.88 (0.74, 1.04)
Secondary	0.76 (0.53, 0.82)*		0.76 (0.63, 0.92)*
Higher	0.38 (0.22, 0.80)*		0.48 (0.32, 0.70)*
1st			1
2nd	3.06 (2.25, 3.93)*		4.06 (3.35, 4.93)*
3rd	4.04 (3.35, 4.83)*		4.55 (3.72, 5.55)*
No	1		1
One	1.08 (1.02, 1.55)*		1.04 (0.99, 1.09)
More than one	1.06 (1.01, 1.83)*		1.16 (1.07, 1.26)*
Marital status			
Living with partner			1
Not living with partner	1.22 (1.11, 1.28)*		1.34 (1.09, 1.65)*
Mass media exposure			
Not at all	1.21 (0.86, 1.26)		
Less than once/week	1.05 (0.27, 1.08)		
At least once/week	1.01 (0.96, 1.15)		
Everyday	1		
No			
Yes	1.01 (0.79, 1.14)		
Unimproved	1.13 (1.01, 1.76)*		1.03 (0.78, 1.15)
Improved	1		1
Unimproved	1		1
Improved	1.23 (1.01, 1.36)*		0.87 (0.74, 1.21)
Clean	1		1
Solid	1.56 (1.50, 1.82)*		1.12 (0.73, 1.26)
<=2	1		
3–4	0.22 (0.87, 0.96)*		0.92 (0.83, 1.98)
>=5	1.07 (0.03, 1.15)		0.88 (0.84, 1.93)
Poorest	1.94 (1.85, 2.04)*		1.82 (1.38, 2.42)*
Poorer	1.64 (1.57, 1.72)*		1.75 (1.34, 2.29)*
Middle	1.50 (1.44, 1.56)*		1.40 (1.09, 1.80)*
Richer	1.28 (1.23, 1.33)*		1.44 (1.15, 1.81)*
Richest	1		1
<=30 min	0.54 (0.21, 0.75)*		0.81 (0.88, 1.97)
> 30 min	0.62 (0.42, 0.78)*		0.85 (0.80, 1.98)
On promises	1		1
Big problem	1.06 (1.04,1.09)*		1.08 (0.82, 1.09)
Not a big problem	1		1
Urban		1.28 (1.17, 1.31)*	1.09 (0.03, 1.15)
Rural		1	1
West Africa		2.89 (2.78, 3.90)*	5.49 (4.67, 6.46)*
Central Africa		1.71 (1.53, 3.95)*	1.92 (1.47, 2.52)*
South Africa		2.09 (0.99, 1.21)	0.59 (0.27, 1.26)
East Africa		1	1
Model fit statistics			
AIC	255,191.73	256,025.317	251,559.27

*p-value <0.05.

## 4 Discussion

The pooled prevalence of anemia was 51.26% among pregnant women in SSA. This is higher than the global (37%) and continental (46.7%) estimates of prevalence of anemia among pregnant women. This shows that anemia is a severe public health problem among pregnant women in SSA.

Pregnant women who have attended secondary and higher education had lesser odds of being at higher levels of anemia as compared to those who have not attended education. This is inline the finding in study done in Ethiopia (19), SSA (20) and Nepal (21). This can be due to the fact that attending school often leads to increased knowledge and awareness about proper nutrition and health practices. Women who have received education are more likely to have access to information about balanced diets and the importance of consuming iron-rich foods. Additionally, attending school may also provide opportunities for women to engage in physical activities, which can contribute to better overall health. Furthermore, education can empower women to make informed decisions about their own health and seek appropriate medical care when needed (22–24).

Pregnant women who were in the old age groups were less likely to be at higher levels of anemia than adolescent pregnant women. This is in line to the finding in study done in Ethiopia (25), East Africa (26) and Uganda (27). It's worth noting that older women may have accumulated more life experiences, including a better understanding of nutrition and healthcare. Older pregnant women may be more likely to follow recommended prenatal care guidelines, including proper nutrition and iron supplementation, which can contribute to a lower risk of anemia during pregnancy (23, 28). Additionally, older women may have a higher baseline iron stores due to a longer period of exposure to dietary sources of iron throughout their lives.

Pregnant women not living with their partner had greater odds of being at higher levels of anemia as compared with those living with their partner. This finding is supported by study conducted in East Africa (26). Living with a partner may provide increased social and emotional support. Emotional well-being and reduced stress can positively impact overall health, including nutritional status. Pregnant women living with their partners may have better financial stability and access to resources. A stable financial situation can contribute to a more nutritious diet, including foods rich in iron and other essential nutrients. Women living with their partners may have better access to healthcare resources, including prenatal care, which is crucial for monitoring and managing health during pregnancy. Regular prenatal check-ups can help identify and address anemia early on. Shared living arrangements may influence dietary habits. Pregnant women living with their partners may be more likely to have access to a variety of foods, including those that are iron-rich, leading to a healthier and more balanced diet. The stress and emotional strain associated with being pregnant and not living with a partner might contribute to lifestyle factors that can impact health. Stress can affect appetite, dietary choices, and overall well-being, potentially contributing to anemia (29, 30).

The odds of being at higher levels of anemia is greater for pregnant women who have given birth to more than one births in the last five years before survey compared to women who gave no birth. This is in line to the finding in studies done in Ethiopia (19) and Bangladesh (31). This could be because pregnancies that happen too soon after one another may leave the body unable to recuperate completely, which could result in the body losing vital nutrients like iron. The body requires time to heal and restore the nutrients that are depleted during pregnancy and delivery. Given that iron is an essential component of red blood cells, this could lead to anemia (32).

Also, frequent pregnancies can lead to increased blood loss during childbirth. Women who have shorter intervals between pregnancies may not have enough time for their bodies to fully restore their blood volume and iron stores before the next pregnancy. This can further contribute to the development of anemia (33).

The odds of being at higher levels of anemia were greater for pregnant women in second and third trimester as compared to those in first trimester. This is in agreement to finding in studies done in Bangladesh (31) and SSA (20). This is due to the fact that the mother's body needs more iron in the latter half of her pregnancy in order to produce more red blood cells and sustain the fetus's rapid growth in the second and third trimesters. Additionally, the increased risk of anemia in the second and third trimesters may be explained by the larger maternal plasma volume increments in relation to red cell mass.

The likelihood of having worse anemia was higher for women in the poorest, middle-class, rich, and poor households than for those in the wealthiest homes. These results were also seen in studies conducted in Mali (34), East Africa (35) and SSA (36). This could be due to the fact that women from lower-income households are less able to pay for preventive and curative medical care as well as diverse, iron-rich foods. One consequence of not being able to pay for medical treatment for their illnesses could be anemia (37, 38). Additionally, women who reside in lower-income households are more vulnerable to anemia-causing infectious illnesses and malnutrition (39, 40).

Lack of information on certain proximal causes of anemia, such as dietary variety, HIV, malaria, and parasite infection, may have had an impact on the reported result. Because of the cross-sectional form of the study, determining the temporal association is much more difficult.

## 5 Conclusion

In Sub-Saharan Africa, anemia is a severe public health concern for pregnant mothers. The percentages of those who were moderately and severely anemic were almost one-fourth (26.8%) and 1.53%, respectively. Factors associated with anemia change on an individual and community levels. When developing and implementing strategies for the prevention and control of anemia, it is imperative to take into account the individual and community circumstances.

Anemia was substantially correlated with age, education, marital status, number of births within the previous five years, pregnancy trimester, wealth index, and region of residence.

It is important to address these factors and provide support to women in order to prevent and manage anemia in this population. Adolescent females, as well as those who were not enrolled in school should receive extra consideration when developing programs to prevent and control anemia. Programs for the prevention and control of anemia should incorporate the economic and educational empowerment of women.

## Data Availability

Publicly available datasets were analyzed in this study. This data can be found here: http://dhsprogram.com.
